# Post mTBI fatigue is associated with abnormal brain functional connectivity

**DOI:** 10.1038/srep21183

**Published:** 2016-02-16

**Authors:** Love Engström Nordin, Marika Christina Möller, Per Julin, Aniko Bartfai, Farouk Hashim, Tie-Qiang Li

**Affiliations:** 1 Department of Diagnostic Medical Physics, Karolinska University Hospital Huddinge, Stockholm, Sweden; 2 Department of Clinical Science, Intervention and Technology, Division of Medical Imaging and Technology, Karolinska Institute, Stockholm, Sweden; 3 Department of Clinical Sciences, Danderyd Hospital, KIDS, Karolinska Institute, Solnavägen 1, 171 77 Solna, Sweden; 4 Centre for Clinical Research, Sörmland, Uppsala University, Uppsala, Sweden; 5 Neurological Rehabilitation Clinic, Stora Sköndal Foundation, Stockholm, Sweden; 6 Department of Radiology, Karolinska University Hospital, Stockholm, Sweden

## Abstract

This study set out to investigate the behavioral correlates of changes in resting-state functional connectivity before and after performing a 20 minute continuous psychomotor vigilance task (PVT) for patients with chronic post-concussion syndrome. Ten patients in chronic phase after mild traumatic brain injury (mTBI) with persisting symptoms of fatigue and ten matched healthy controls participated in the study. We assessed the participants’ fatigue levels and conducted resting-state fMRI before and after a sustained PVT. We evaluated the changes in brain functional connectivity indices in relation to the subject’s fatigue behavior using a quantitative data-driven analysis approach. We found that the PVT invoked significant mental fatigue and specific functional connectivity changes in mTBI patients. Furthermore, we found a significant linear correlation between self-reported fatigue and functional connectivity in the thalamus and middle frontal cortex. Our findings indicate that resting-state fMRI measurements may be a useful indicator of performance potential and a marker of fatigue level in the neural attentional system.

The annual incidence of hospital-treated mild traumatic brain injury (mTBI) has been estimated to be 100–300 per 100.000 in the industrialized world and over 600 per 100,000 when including those who do not seek emergency medical care^1^. Chronic fatigue is one of the most commonly reported and long-lasting post concussion symptoms^2^,^3^,^4^. Enhanced sensitivity to effort and limited endurance for sustained physical and mental activities are the main characteristics of central fatigue^5^. The concept of fatigue appears deceptively simple, but researchers and clinicians do not as yet have a commonly accepted objective measure for it. Like pain, fatigue is a private and subjective experience, which is largely measured through self-report. Cognitive fatigability has recently been proposed as a possible objective assessment of fatigue, and can be assessed as fluctuations and decline in performance of a vigilance task as a function of time^3^. Its clinical applicability in mTBI as a neuropsychological measure of cognitive aspects of fatigue has been demonstrated^3^.

In the past, direct evidence of structural changes underlying chronic fatigue in mTBI patients has been difficult to obtain using conventional imaging modalities such as T1- and T2-weighted magnetic resonance imaging (MRI) and computed tomography (CT)^6^,^7^, which helped proliferate the view that mTBI did not lead to manifest neuronal pathology. Recently, there has been a dramatic change in understanding the pathological consequences of mTBI, as a result of the substantial increase in the number of individuals diagnosed with chronic traumatic encephalopathy amongst recently deceased athletes^8^. Numerous neuroimaging studies of mTBI have conducted in the last decade using different imaging modalities including diffusion tensor imaging (DTI), functional MRI (fMRI), and the metabolic imaging methods such as positron emission tomography and magnetic resonance spectroscopy (MRS), where each targets different aspects of the neuropathology involved in mTBI. DTI provides a highly sensitive measure for detection of primary changes in the microstructure of white matter tracts and has been proved to be a valuable tool to identify diffuse axonal injury (DAI) after mTBI^9^,^10^. fMRI enables the measurement of the functional response of the brain to different stimuli, or of functional connectivity through intrinsic activity in the resting-state^11^. MRS can selectively measure disturbance of the brain metabolites in mTBI^12^.

Remarkably, these techniques have been quite successful in mTBI studies and provided important new lines of evidence suggesting that a single concussion can result in lifetime impairment for some individuals and the neuropathology may be present long after the concussion^9^,^10^,^12^,^13^,^14^,^15^,^16^,^17^. Particularly, the large number of resting-state fMRI studies in the last few years have led to some interesting findings regarding abnormal brain functional connectivity in mTBI patients. Reduced connectivity across multiple nodes within the default mode network (DMN) has been reported in the semi-acute and chronic phases of mTBI^17^,^18^,^19^,^20^,^21^,^22^,^23^,^24^,^25^. The functional connectivity abnormalities remained even 4 months post-injury. Resting-state fMRI studies of mTBI patients with post-concussion syndrome have also revealed reduced functional connectivity at the chronic phase in other networks aside from the DMN, including the thalamic network^26^, motor-striatal network^15^, frontal regions^13^, task positive and salience networks^14^. Disrupted functional connectivity in multiple sensory and cognitive networks has also been observed in mtBI patients with persistent post-concussive syndrome^16^,^24^.

With the development of advanced neuroimaging methods substantial progress has been made in improving our understanding of the neuropathology of post mTBI patients with chronic fatigue. However, the interrelationship between fatigue and abnormal brain functional connectivity in mTBI remains elusive. As outlined above, we can attribute this to the following factors:

1. Fatigue is not a well-defined concept and lacks objective measure.
2. Results of fMRI studies of mTBI patients showed a variety of abnormal functional connectivity in different networks.
3. Resting-state fMRI methodology is lacking quantitative metrics, rendering it difficult to compare the measurements from different studies and time points.

Furthermore, resting-state fMRI data are currently analyzed either by using a model-driven approach, such as the use of predefined seed region of interests (ROIs) or a data-driven approach, such as the independent component analysis (ICA). The former requires a priori knowledge of the anatomical model, which is usually not available or biased. The later requires the input of the number of independent components, which is difficult to estimate a priori and can influence the derived results substantially^27^,^28^,^29^,^30^.

The main goal of this study is to investigate the interrelationship between post mTBI fatigue and brain functional connectivity, which has not been explicitly assessed in previous studies of mTBI. We developed a voxel-based quantitative data-driven analysis (QDA) method for the analysis of resting-state fMRI data, which derives two metrics named as connectivity count index (CCI) and connectivity strength index (CSI) allowing for direct comparison of functional connectivity measurements between different subjects and at different time points^31^. We employed this QDA method to analyze the resting-state fMRI measurements conducted in a group of mTBI patients diagnosed with post-concussive syndrome and a group of matched healthy controls. To study the dynamics of brain functional connectivity in relation to fatigue we performed resting-state fMRI measurements and fatigue assessment of the participants both before and after a sustained continuous psychomotor vigilance task (PVT) to evoke cognitive fatigue and subjective fatigue change in mTBI patients.

## Results

### Characterization of the participants

The mTBI patients reported significant higher general fatigue (Fatigue Severity Scale, FSS) (median_pat_ 5.8, range 4.8–6.7) than the healthy controls (median_con_ 2.6, range 1.8–3.0) p ≤ 0.001. There was no significant difference between patients and controls regarding self-rated sleep quality (Pittsburgh Sleep Quality Index, PSQI) (median_pat_ 9.5, range 2.0–14.0 vs. median_con_ 5.0 range 3.0–10.0, p = 0.063) but the patients slept longer than the controls the night before the MRI scanning (median_pat_ 8.0 hours, range 6.0–10.0 hours vs. median_con_ 6.0 hours range 4.0–8.0 hours, p = 0.013). There was no significant difference between patients and controls regarding tea and coffee consumptions before the MRI investigation. The body mass index (BMI) was approximately the same for the two groups.

There was no significant difference in self-rated current fatigue (Visual Analogue Scale of fatigue, VAS-f) between patients and controls before MRI scanning (median_pat_ = 5.8, range 2.3–8.1 vs. median_con_ 3.0 range 0.9–7.7, p = 0.143), but directly after the PVT performance the patients scored significantly higher for current fatigue than the controls (median_pat_ 8.0, range 5.5–9.7 vs. median_con_ 4.0 range 1.3–6.9, p ≤ 0.001). There was a significant increase in VAS-f for the mTBI patients after the PVT (p = 0.008), whereas the increase in healthy controls was not significant (p = 0.508). Compared to the controls, the mTBI patients displayed a significant decrease and less stability in cognitive performance in the PVT as seen in the significantly longer mean reaction time, RT_mean_ (577 ± 162 ms versus 414 ± 29 ms, p = 0.006) and higher standard deviation, RT_std_ (110 ± 47 ms versus 61 ± 14 ms, p = 0.006). The mean VAS-f after the PVT was significantly correlated with both the RT_mean_ (r = 0.606, p = 0.005) and the RT_std_ (r = 0.611, p = 0.004).

There were no structural pathological findings from the evaluation of the clinical MRI scans to exclude any subject. However, clinical MRI scans showed multiple small hemorrhagic lesions distributed in the left hemisphere in the parasagittal frontal lobe and splenium as well as a suspected lesion in the pons on the right side for one patient included in this study. These findings were considered to be consistent with diffuse axonal injury. This patient did meet all inclusion criteria for mTBI and did not deviate on the PVT.

### The effects of PVT on brain functional connectivity

**Figure 1:**
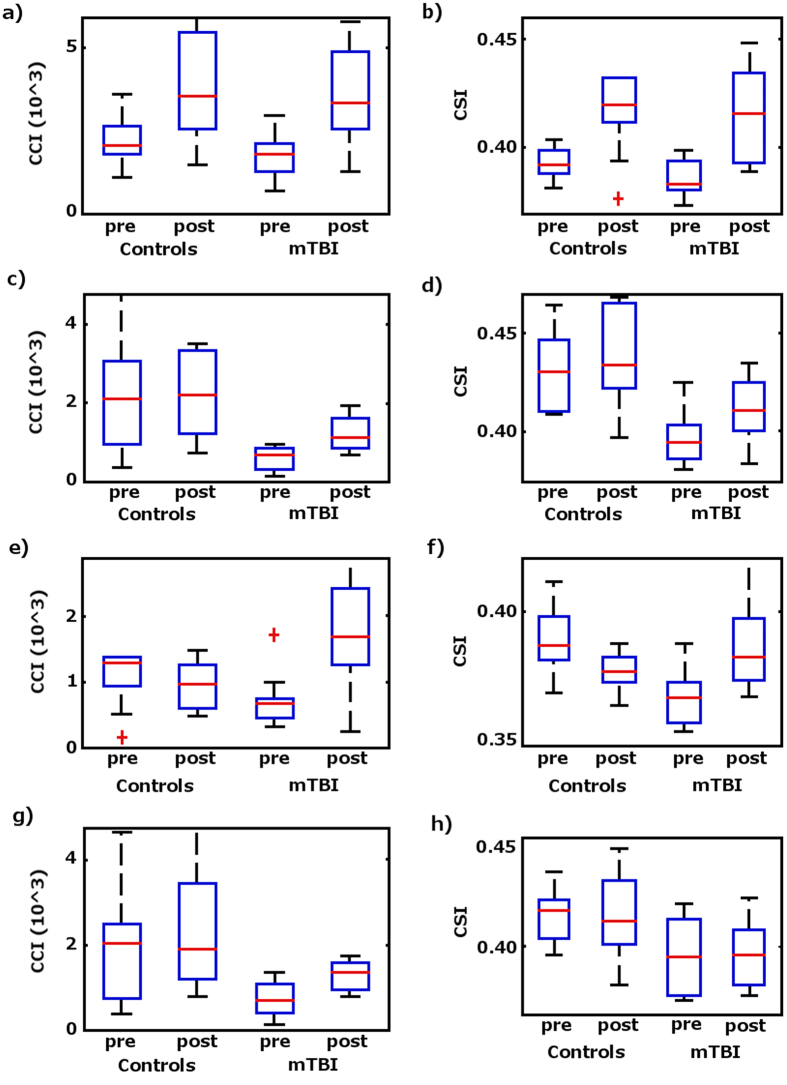
Boxplots of the ROI averages of CCI and CSI for the brain regions with significant effects (p ≤ 0.05) of the 2 fixed factors (the PVT and subject group) and their interactions as assessed with 3-way ANOVA method. (**a**) The effect of the PVT on CCI; (**b**) The effect of the PVT on CSI; (**c**) CCI difference between mTBI patients and healthy controls; (**d**) CSI difference between mTBI patients and healthy controls; (**e**) The interaction effect of CCI showing that mTBI patients and healthy controls are affected differently by the PVT performance; (**f**) The interaction effect of CSI showing that mTBI patients and healthy controls are affected differently by the PVT. (**g**) Average CCI for ROIs with significant correlation with VAS-f; (**h**) Average CSI for ROIs with significant correlation with VAS-f. The central marks of the boxplots are the median, the edges of the box are the 25th and 75th percentiles, the whiskers extend to the most extreme data points considered to be not outliers, and the outliers are plotted individually as red crosses.

**Figure 2:**
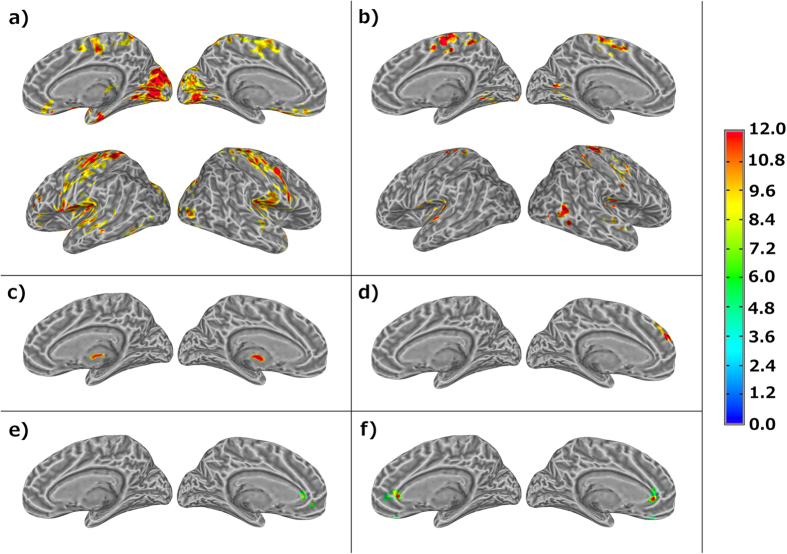
Summary of F-score results from the 3-way ANOVA analysis of CCI and CSI data to depict the brain regions of statistically significant differences (p ≤ 0.05) in functional connectivity associated with the 2 fixed factors (the PVT and subject group) and their interaction. (**a**) The brain regions with statistical significant change in CCI before and after PVT for all participants; (**b**) The brain regions with statistically significant change in CSI before and after PVT for all participants; (**c**) The brain regions with statistically significant CCI difference between mTBI patients and healthy controls; (**d**) The brain regions with statistically significant CSI difference between mTBI patients and healthy controls; (**e**) The brain regions with statistically significant interaction effect of two main factors on CCI. That is the brain areas where CCI of the mTBI patients and healthy controls are affected differently by the PVT performance; (**f**) The brain regions with statistically significant interaction effect of two main factors on CSI. That is the brain areas where CSI of the mTBI patients and controls are affected differently by the PVT performance.

Figures 1 and 2 depict the results obtained from the 3-way ANOVA of the functional connectivity indices data (CCI and CSI). As shown in Fig. 2a,b, both CCI and CSI are significantly (p ≤ 0.05) influenced by the PVT task. The brain areas that show a significant change in CCI are more than 5 times bigger than the areas with a significant change in CSI (see Table 1). Region of interest (ROI) averages (Fig. 1) demonstrate that both CCI and CSI are increased after the PVT. As detailed in Table 1, PVT induced CCI and CSI increase in a number of functional networks involving both motor-sensory, attention and executive control. Comparing mTBI patients with the healthy controls, there are significant (p ≤ 0.05) differences in CCI and CSI in thalamus and medial frontal gyri (MFG), respectively (see Fig. 2c,d). The ROI averages (see Fig. 1) for the brain regions with significant group difference indicate that the healthy controls have more extensive functional connectivity networks (higher CCI) in the thalamus and stronger functional connectivity (higher CSI) in MFG both before and after the PVT task.

The CCI and CSI in the dorsal anterior cingulate (ACC) depict significant (p ≤ 0.05) interaction effects between the two fixed factors (see Fig. 2e,f and Table 1). As shown in Fig. 1e,f, the vigilance stimuli affect the functional connectivity of the mTBI patients and healthy controls differently. For the healthy controls both CCI (1371 ± 1148 versus 729 ± 402) and CSI (0.388 ± 0.013 versus 0.369 ± 0.011) are slightly reduced after the PVT, whereas the CCI (962 ± 387 versus 1770 ± 1053) and CSI (0.378 ± 0.007 versus 0.390 ± 0.021) results for the mTBI patients show the opposite trend.

**Figure 3:**
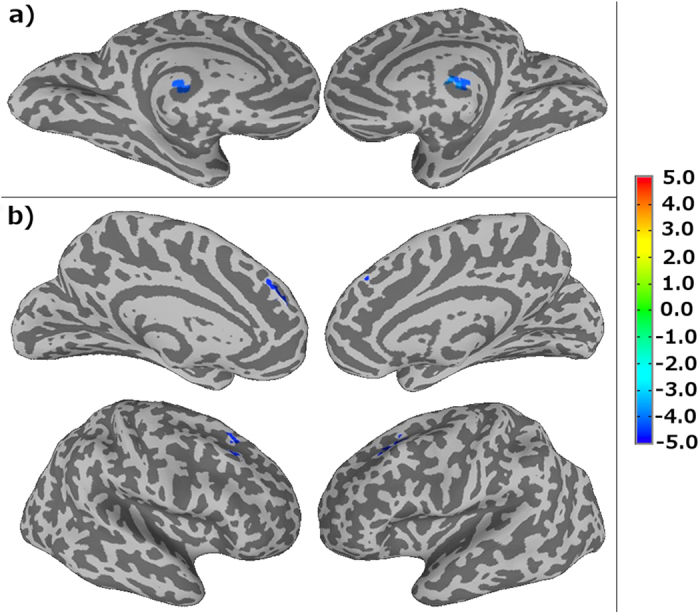
Results obtained from the linear regression analysis of functional connectivity as a function of the self-reported fatigue. (**a**) Brain regions with significant (p ≤ 0.05) correlation between CCI and VAS-f. (**b**) Brain regions with significant (p ≤ 0.05) correlation between CSI and VAS-f.

**Figure 4:**
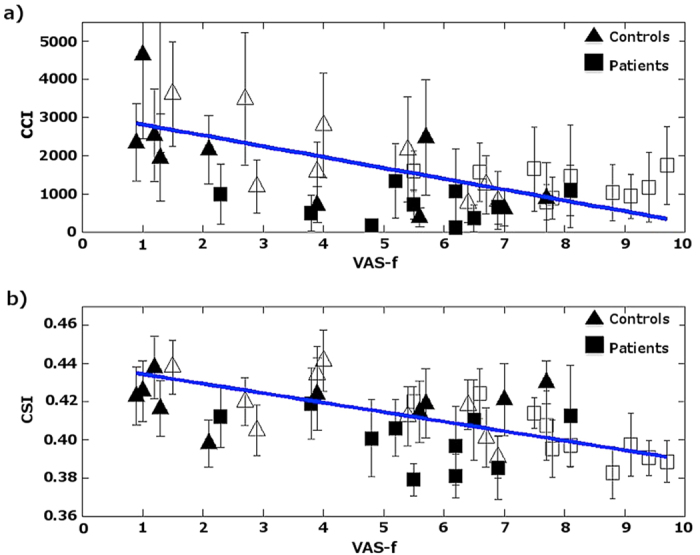
Scattered plot of the ROI averages of functional connectivity for the brain regions that have significant (p < 0.05) linear correlation with self-reported fatigue. (**a**) ROI averages of CCI as a function of VAS-f for all participants. (**b**) ROI averages of CSI as a function of VAS-f for all participants. The error bars denote the standard deviation of the different voxels within the detected ROI. The filled and blank symbols represent the measurements before and after the PVTs, respectively.

**Figure 5:**
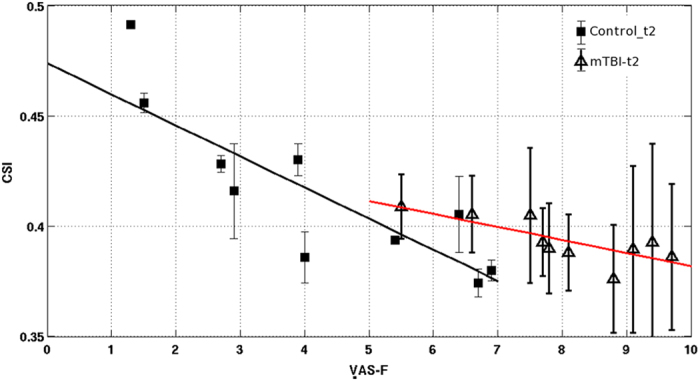
Scattered plot of the ROI averages of functional connectivity for the brain regions that have significant (FWER, p ≤ 0.05) linear correlation with self-reported fatigue. (**a**) ROI averages of CSI as a function of VAS-f for the control subjects after PVT (control_t2). (**b**) ROI averages of CSI as a function of VAS-f for mTBI participants after PVT (mTBI_t2). The error bars denote the standard deviation of the different voxels within the detected ROI. The black and red lines represent the linear regression estimates for the controls and mTBI patients, respectively.

The RT_mean_ measured during the PVT is not significantly correlated with the change in functional connectivity indices. However, the self-reported fatigue, VAS-f, is significantly correlated (p ≤ 0.05) with CCI in thalamus and CSI in MFG (see Fig. 3), respectively. The brain regions showing significant correlations between resting-state functional connectivity and VAS-f overlap largely with the brain areas found to show significant group difference between mTBI patients and healthy controls as identified by 3-way ANOVA. As shown in Fig. 4, CCI in the thalamus is negatively correlated with VAS-f (r = −0.57, p = 0.0002) and CSI in MFG is negatively correlated with VAS-f (r = −0.58, p = 0.0002), when data from all participants were pooled together. However, when data for each subject group were analyzed separately, only CSI measured after PVT in MFG is negatively correlated with VAS-f both for healthy controls and mTBI patients (see Fig. 5). As detailed in Table 2, in MFG there is a trend (but not statistically significant) of negative correlation between CSI measured before PVT and VAS-f. Similar trend was also observed for CCI measured in healthy controls.

### The effects of CC threshold value on the statistical results of CCI and CSI

**Figure 6:**
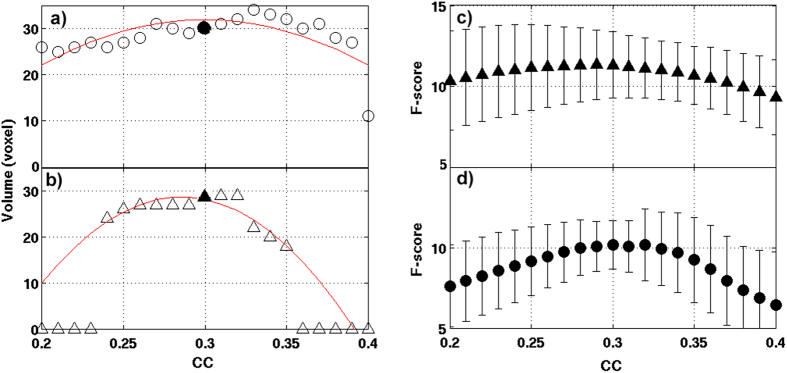
The effect of CC threshold on results from the 3-way ANOVA modeling of the derived CCI and CSI maps is illustrated. (**a**) The detected brain volume with statistically significant subject group difference in CCI (FWER, p ≤ 0.05) as function of CC threshold value; (**b**) The detected brain volume with statistically significant subject group difference in CSI (FWER, p ≤ 0.05) as function of CC threshold value; (**c**) The ROI average F-score for the detected brain volume with statistically significant subject group difference in CCI (FWER, p ≤ 0.05) as function of CC threshold value; (**d**) The ROI average F-score for the detected brain volume with statistically significant subject group difference in CSI (FWER, p ≤ 0.05) as function of CC threshold value; The curves in (**a**,**b**) denote a 2^nd^-order polynomial non-linear curve fittings to the detected brain volumes as function of CC threshold values. The error bars in (**c**,**d**) denote the standard deviation of the different voxels within the detected ROI.

As shown in Fig. 6, the ANOVA results of CCI and CSI are relatively insensitive to the CC threshold value between 0.26 ≤ CC ≤ 0.37. The selection of a threshold value CC = 0.3 is quite optimal in term of achieving the maximum statistical sensitivity as measured by the ROI average F-score and the detected brain volume.

## Discussion

The major novel findings of the present study are the following: (1) Post mTBI fatigue in the chronic phase is significantly correlated with abnormal brain functional connectivity in the thalamus and MFG; (2) A 20 min PVT is sufficient to invoke statistically significant differences in mental fatigue and specific functional connectivity changes in mTBI patients; (3) Resting-state fMRI combined with QDA is a sensitive method for detecting abnormalities in brain functional connectivity of mTBI patients.

The observation of aberrant functional connections of the thalamus and MFG in mTBI is not surprising, because various studies have shown that thalamus is one of the “rich club members” of the brain’s functional connectivity and is an important hub for information flow control between different subcortical areas and the cerebral cortex^32^,^33^,^34^. The thalamus plays a key role in motor control, processing of different sensory signals, and maintaining homeostasis of autonomic processes. Subtle thalamic injuries resulting in functional hypo-activity are expected to have substantial effect on the efficiency for mental operations.

Recent studies have identified MFG as the site of convergence of dorsal and ventral attention networks and MFG plays a pivotal role in endogenous and exogenous attention controls^34^,^35^. Reduced CSI of MFG in mTBI patients implicates a lowered intrinsic functional synchronization within the attention networks, which is in line with previous neuropsychological findings that fatigue was related to reduced processing speed^36^, higher order attention^37^, and in executive functions^38^. It is plausible that subtle brain structural injuries in mTBI can lead to disruption of functional networks involving thalamus and MFG and lowered efficiency in the performance of complex cognitive tasks. The patients have to make more mental effort to maintain the performance of a complex task, such as the continuous PVT used in the current study, which can result in up-regulating of functional connectivity in thalamus and MFG. This may eventually lead to the exhaustion of the necessary resources for the execution of the task and cognitive fatigue. It should be pointed out that the increase of brain functional connectivity to the thalamus has previously been reported for mTBI patients in the sub-acute phase, using a seed-based analysis^39^.

To date it is still not clear which biological substrate underlie fatigue in normal individuals. Therefore, the understanding of the pathophysiology of post-mTBI fatigue is also in the development stage. Brain imaging studies in the past decade have contributed to the rapid growth in knowledge of our understanding of the probable pathophysiology for post mTBI fatigue. As discussed above, there is emerging evidence to support the notion that mild brain trauma can manifest neuronal dysfunction and result in diffuse axonal damage, and changes in neurotransmission^40^.

By taking advantage of a QDA approach and employing PVT to invoke sustained cognitive load, we were able to detect robust brain functional connectivity changes in mTBI patients in relation to their alteration of fatigue behavior, which is likely to reflect the subtle structural injuries of the involved brain regions. As discussed above, the PVT resulted in not only significant performance variability in mTBI patients, but also significant self-reported “current” fatigue direct after the task. Comparing the functional connectivity results measured before and after PVT, it is clear that PVT induced overall functional connectivity increases in a number of brain functional networks including sensorimotor cortices, visual cortex, basal ganglia, and fronto-parietal attention network. The up-regulating of resting-state functional connectivity directly after PVT was achieved largely by recruitment of additional components as evidenced by the substantially larger volume increase in CCI relative to CSI.

With a QDA approach, we can conduct direct correlation analysis between fatigue assessments and functional connectivity indices to identify the brain regions where fatigue complains are associated with the change of functional connectivity. It is interesting that the brain regions showing significant differences in functional connectivity between the mTBI patients and healthy controls overlaps largely with the ROIs where significant linear correlations between fatigue and functional connectivity indices exists. This implies that the brain regions with disrupted functional connectivity and group differences play a direct role in executing the task load giving rise to fatigue and cognitive fatigability. Although the correlation results do not necessarily imply causality, the negative correlation coefficients between fatigue and functional connectivity indices in thalamus and MFG indicate that fatigue is accompanied with local resource depletion within the involved functional networks^41^. The correlation results of this study are quite consistent with the findings from a previous ASL fMRI study of healthy volunteers using a similar PVT^42^. It was reported that PVT stimuli activated the thalamus and a fronto-parietal attention network involving right MFG in addition to the basal ganglia and sensorimotor cortices. The regional CBF in the MFG attention network was negatively correlated with performance decline. The correlation results display the critical role of the thalamic and attention networks in mediating cognitive fatigability. Furthermore, resting-state functional connectivity in the thalamus and MFG are predictive of subjects’ subjective fatigue and resting-state fMRI measurements may be a useful indicator of performance potential and a marker of fatigue level in the neural attention system.

The change of functional connectivity in the dorsal anterior cingulate cortex (ACC) before and after the sustained PVT task has a significant interaction effect between subject group and PVT task, but is not significantly correlated with self-reported fatigue and cognitive fatigability. Both CCI and CSI were down regulated for the healthy controls after the performance of PVT, whereas the opposite trend was observed for the mTBI patient group. ACC is generally active in task settings that are experienced as cognitively difficult and is importantly involved in signaling the occurrence of conflicts in information processing^43^. The function of ACC has been hypothesized to monitor conflict as an index of difficulty, evaluate effort demand and outcome, and thereby trigger compensatory adjustments in cognitive control^43^,^44^,^45^. A plausible way of reconciling the observed dynamics of brain functional connectivity with respect to the performance of PVT is to consider that ACC is only responsible for the monitoring of task difficulty and signaling effort adjustment but not the execution of the task itself. Therefore, the change of functional connectivity in ACC is not correlated with fatigue itself. The down-regulating trend in the healthy controls is likely due to the skill learning effect leading to signaling of reduced effort, while the up-regulating change in the mTBI patients is probably because of the reduced efficiency resulting in signaling of increased effort demand and regulation of effort 46.

The PVT task augmented the differences in self-reported fatigue between mTBI patients and controls. Self-reported fatigue is a multidimensional concept involving both physiological and psychological aspects. Aside from cognitive performance, it also includes secondary effects of factors, such as mood, depression and sleep quality. On the other hand, cognitive fatigability reflects the inability to maintain performance during a sustained task of complex information processing. Three well-defined cognitive domains are predominantly engaged: attention, executive, and psychomotor functions. Cognitive fatigability can be objectively measured by quantifying performance deterioration over time. Therefore, RT_STD_ is a better measure than RT_mean_ for the assessment of cognitive fatigability. This explains also why the self-reported fatigue (FSS and VAS-f) has a higher correlation with RT_STD_. In line with findings from previous studies^3^, the moderate correlation between the self-reported fatigue and RT_STD_ supports the view that self-reported subjective fatigue overlaps partially with cognitive fatigability. The degree of overlap is not only dependent on the characteristics of the patients but also on the task design used for assessing cognitive fatigability.

For the continuous vigilance task used in the present study the individuals admittedly need to be continuously attentive and its successful of performance also requires cooperation between other domains such as vigilance, selective attention, decisional ability, inhibition control of an overlearned response and speedy motor response to the visual cue of numbers. The fact that the task can differentially induce cognitive fatigability in mTBI patients implies that maintaining performance in the continuous PVT task demands deliberate control of behavior with high sensitivity to cognitive impairment and attention deficits. It has been suggested that disturbances in motor function, higher order attention, and executive control can all be associated with fatigue-related deteriorations^2^,^3^,^4^. The relative contribution of these factors to cognitive fatigability is determined by the characteristics of the test.

The methodological strengths of this study are the followings: (1) we take advantage of the sustained PVT that induced significant fatigue and cognitive fatigability in mTBI patients; (2) we combined a QDA approach with resting-state fMRI, which allows for direct comparisons of functional connectivity of subject groups measured before and after PVT. This study has a number of limitations. The number of participants is relatively small. A Larger sample size is likely to produce more robust results from the linear regression analysis of individual subject group. We attempted to exclude confounding factors, such as the presence of psychiatric conditions, history of earlier brain pathology, and substance abuse. However, there is still a lack of full neuropsychological characterization of the participants. Another limitation is that the mTBI patients have a high variability in age and the length of time between the trauma incident and the current assessment. To compensate for this we recruited a control group matched for age, gender and years of education. Overall, the results show promise for further investigations regarding the behavioral correlates of changes in brain functional connectivity. However these results should be further verified in larger studies.

## Materials and Method

### Participants

The study sample consisted of 10 mTBI patients (m/f 5/5). The time between trauma and assessment exceeded at least 6 months, with a median of five years, (range 0.5–9 years). The diagnosis of mTBI was defined according to the American Congress of Rehabilitation Medicine (1993); traumatically induced physiological disruption of brain function, as manifested by at least one of the following^47^:

- a period of loss of consciousness (LOC) of up to 30 minutes;
- any loss of memory for events immediately before or after the accident but with a duration of less than 24 hours;
- any alteration of mental state at the time of accident; score on the Glasgow Coma Scale (GCS) administered after 30 min from trauma between 13 and 15;
- presence of focal neurological deficit that may or may not have been transient.

For study inclusion persisting complaints of fatigue, defined as a mean score of 4 or above on the fatigue severity scale (FSS), were mandatory.

Patients were excluded if the duration of loss of consciousness was uncertain (e.g. in combination with alcohol intoxication), if CT-scan indicated dementia, hydrocephalus or subdural hematoma, or if the patient suffered from seizures or a severe psychiatric disorder. The patients were aged between 20 and 52 years with a mean age of 37.5 years (SD 11.2), and mean educational length of 13.1 y (SD 1.6). Three patients suffered from traffic accidents, five from fall accidents. One patient suffered from a bicycle accident and one patient had a riding accident. An experienced neuroradiologist evaluated the clinical MRI scans for all participants.

A healthy control group (n = 10, m/f 5/5) matched for age, gender and years of education was also recruited for the study. For the control group the mean age was 36.9 y (SD 11.0, range 20–47) and the mean educational length was 13.4 y (SD 2.0).

### Characterization of the participants

Self-rated general fatigue was measured with the fatigue severity scale (FSS). A mean score over 4 indicates fatigue^48^,^49^. The self-rated current fatigue level (“How fatigued do you feel right now”) was assessed using a visual analog scale of fatigue (VAS-f). The VAS-f ranges from 0 corresponding to no fatigue, to 10 corresponding to the worst possible fatigue. Sleep quality was measured with a Swedish version of the Pittsburgh Sleep Quality Index (PSQI)^50^.

Medications, tobacco, coffee and tea consumptions, food and fluid intakes and the number of hours of sleep were recorded for each participant on the day of examination. The participants were also asked if they had experienced anything that could have significantly affected their responsiveness.

### Ethical Approval and Patient Consents

This study was approved by the regional ethics committee in Stockholm, Sweden, and was carried out in accordance with the approved guidelines. All participants received verbal and written information about the study before signing an informed consent.

### Psychomotor vigilance test

The PVT lasted 20 minutes inside the MRI scanner. The setup for the test included a projector-based visual system with feedback to measure the reaction times (RT) of the participants. The stimulus paradigm was created in E-Prime software (Psychology Software Tools Inc., Pittsburgh, PA). The participants were instructed to press a response button as quickly as they could when a set of zeroes appeared on the screen. The paradigm also included false starts, when the participants were instructed not to press the response button when numbers appeared on the screen.

### Experimental Procedure

All participants completed the self-reported fatigue assessments according the FSS and VAS-f measures before entering the MRI study that lasted for about 66 minutes. The participants also had a 2 minute training session of the PVT before the MRI scans described below. Directly after the MRI scanning the participants rated their level of current fatigue for a second time on the VAS-f.

### MRI Data Acquisition Protocol

The MRI data acquisition was conducted on a whole-body 3T clinical MRI scanner (Magnetom Trio, Siemens Medical Solutions, Erlangen, Germany) equipped with a 32-channel phased-array receiving head coil. All data was acquired at Karolinska University Hospital Huddinge, Stockholm between noon and 5:00pm. The MRI data acquisition protocol included the following scanning sessions: (1) 3-plane localizer; (2) Conventional clinical MRI scans including 3D T1-weighted MPRAGE, SWI, FLAIR, T2-weighted GRE and DWI; (3) The 1^st^ session of 8-minute long resting-state fMRI; (4) a 20-minute long PVT task with simultaneous pseudo-continuous arterial spin-labeling (pCASL) measurements; (5) The 2^nd^ session of 8-min long resting-state fMRI. The resting-state fMRI data were acquired using a gradient-recalled echo 2D echo-planar imaging technique. The main acquisition parameters included the following: TE/TR 35/2000 ms, flip angle = 90°, 34 slices of 3.5 mm thick, FOV = 204 mm, matrix size = 68 × 68, data acquisition acceleration with GRAPPA parallel imaging method (iPAT = 2), and 240 dynamic timeframes.

Foam padding was used to fix each participant’s head carefully in in the head coil and reduce involuntary head motions. During the resting-state fMRI scans the participants were instructed to focus their sight on a white cross onto a black background projected on a screen installed in front of them. The participants were also instructed to not think about anything specific during the resting-state fMRI scans.

### Resting-state fMRI data processing

The resting-state fMRI datasets underwent a preprocessing procedure, which was performed with AFNI (http://afni.nimh.nih.gov/afni) and FSL (http://www.fmrib.ox.ac.uk/fsl) programs with a bash wrapper shell^30^,^51^. The first 5 timeframes in each data set were removed to ensure signal steady state. After temporal de-spiking, six-parameter rigid body image registration was performed for motion correction. The average volume for each motion-corrected time series was used to generate a brain mask to minimize the inclusion of the extra-cerebral tissues. Spatial normalization to the standard MNI template was performed using a 12-parameter affine transformation and mutual-information cost function. During the affine transformation the imaging data were also re-sampled to isotropic resolution using a Gaussian kernel with 4 mm full width at half maximum (FWHM). Nuisance signal removal was performed by voxel-wise regression using 14 regressors based on the motion correction parameters, average signal of the ventricles and their 1^st^ order derivatives. After baseline trend removal up to the third order polynomial, effective band-pass filtering was performed using low-pass filtering at 0.08 Hz. Local Gaussian smoothing up to FWHM = 5 mm was performed using an eroded gray matter mask 30.

Taking advantage of the availability of rapidly expanding computational power, we have developed a framework to compute the voxel-wise cross-correlation coefficient matrix^31^. That is for each voxel inside the brain, we compute the Pearson’s cross-correlation coefficients of the resting-state fMRI time course with that of every other voxel inside the brain. To conduct QDA of the resting-state fMRI data using the computed voxel-wise cross-correlation coefficient matrix, for each voxel, we computed the following two voxel-wise metrics: (1) CSI which is defined as the non-zero mean value of the Pearson’s cross-correlation coefficients ≥ 0.3 for all voxel pairs involving the current voxel in question; (2) CCI which is defined as the number of voxel pairs involving the current voxel in question with the Pearson’s cross-correlation coefficients ≥ 0.3.

### Statistics

We used the Mann-Whitney U-test (questionnaires) and Students t-test (PVT data) to analyze the behavioral data and compare the results between mTBI patients and controls. We used the Wilcoxon signed rank test for comparison within subjects. Correlation coefficients between VAS-f, FSS, RT_mean_, and RT_STD_ were calculated using Matlab (MathWorks, Natick, USA)

For comparison of the functional connectivity (CCI and CSI) results between mTBI patients and healthy controls measured before and after the PVT, we performed voxel-wise 3-way analysis of variance (ANOVA) of the CCI and CSI images using the AFNI program 3dANOVA3 with model type = 5. The group difference between mTBI patients and healthy controls (df = 1) and temporal difference before and after the PVT (df = 1) were modeled as fixed factors. The participants in each group were modeled as a random factor (df = 9). With 3-way ANOVA, we can also assess the interaction between the 2 fixed factors and interrogate how the continuous PVT performance affected the functional connectivity in mTBI patients and healthy volunteers.

To study the correlation between fatigue and brain functional connectivity derived from resting-state fMRI measurements, voxel-wise linear regression analysis was performed with the CSI and CCI image data using the AFNI program, 3dRegAna. To gain sufficient statistical power, the data for all participants acquired before and after the PVT task were first pooled together for the regression analysis. Further regression analyses were also performed for each group separately. It should be noted that age and gender were included as cofactors to exclude possible confounding. The voxel-wise CSI or CCI values for all participants were modeled as a linear function of their corresponding self-reported fatigue measures (VAS-f) or cognitive fatigability in terms of the mean reaction time (RT_mean_) and standard deviation of the RT (RT_STD_)measured during the PVT.

The statistical significance of the 3-way ANOVA and regression results were assessed first by setting an uncorrected voxel-wise threshold at p < 0.01 and then by imposing a minimum voxel cluster size of at least 11 contiguous voxels. The probability of random field of noise producing a cluster of size ≥ 11 was estimated at family-wise error rate (FWER), p < 0.05. This was concluded from the Monte-Carlo simulation result obtained by using the AFNI program, *AlphaSim *+* *. For the simulations we used following essential input parameters: matrix size = 45 × 45 × 54, a gray matter mask based on MNI template, voxel-wise threshold value p < 0.01, 10^6^ iterations, and FWHM = 5.2 mm, which was the estimated average by applying the AFNI program, 3dFWHMx, to the CSI and CCI image data, which was quite close to FWHM = 5 mm used in the final imaging smoothing procedure described above.

To investigate how the CC threshold value affect the statistical analysis of the derived CCI and CSI data, we systematically incremented the CC threshold value from CC = 0.2 to 0.4 with an increment step of 0.01. For each CC threshold value, we performed 3-Way ANOVA modeling of the corresponding CCI and CSI data and evaluated the F-score and brain volume satisfying the statistical significance criteria described above.

## Additional Information

**How to cite this article**: Nordin, L. E. *et al.* Post mTBI fatigue is associated with abnormal brain functional connectivity. *Sci. Rep.* **6**, 21183; doi: 10.1038/srep21183 (2016).

## Figures and Tables

**Table 1:** List of brain regions with significant statistical contrast (p < 0.05, corrected) as identified by voxel-wise 3-way ANOVA of CCI and CCI data and linear regression analysis of CCI and CCI against self-reported current fatigue VAS-f.

Index	Statistical Contrast	ROI	Volume (voxel)	X (mm)	Y (mm)	Z (mm)	Location label
	Group	1	30	−11.5	+7.5	+6.5	L- and R-thalamus
		2	1368	−2.5	+75	+2.0	L- and R-cuneus
		3	844	+24.5	+39.5	+62.5	L-pre/postcentral gyrus
		4	528	−51.5	+7.5	+63.0	R-pre/postcentral gyrus
		5	112	−15.5	−4.5	−17.5	R-parahippocampal gyrus
	PVI task	6	88	−35.5	−4.5	+17.5	R-superior temporal gyrus
		7	69	+0.5	+32.5	−17.5	L-medial frontal gyrus
CCI		8	28	+62.5	−20.5	−5.5	L-inferior frontal gyrus
		9	23	+41	+28	+7	L-middle occipital gyrus
		10	22	+67	+20	+3	L- parahippocampal gyrus
	Interaction	11	20	+4.5	−36.5	+6.5	L-anterior cingulate
		12	13	+52.5	+71.5	−13.5	R-anterior cingulate
	Group	1	29	+0.5	−44.5	+30.5	R- medial frontal gyrus
		2	245	−23.5	+31.5	+54.5	R-postcentral gyrus
		3	163	−19.5	+87.5	−17.5	R-fusiform gyrus
		4	53	−43.5	+23.5	+10.5	R-superior temporal gyrus
		5	35	−15.5	+47.5	+66.5	R-lingual gyrus
CSI	PVT task	6	32	+26.0	+33.5	+56.0	L-postcentral gyrus
		7	30	−47.5	+63.5	−1.5	R-middle temporal gyrus
		8	29	+5.5	−3.0	+49.0	L- and R-cingulate gyrus
		9	21	+44.5	+27.5	+6.5	L-superior temporal gyrus
	Interaction	10	20	−7.5	−32.5	−1.5	L-, R- anterior cingulate

X, Y, and Z represent the peak locations of ROIs in the standard MNI coordinates.

**Table 2:** Summary of linear regression analyses of CCI and CSI against self-reported current fatigue VAS-f.

Index	Dataset	*r*	*p*	Volume (voxel)	X (mm)	Y (mm)	Z (mm)	Location label
	Control-t1	−0.43	0.211					
	Control-t2	−0.61	0.063					
CCI	mTBI-t1	0.08	0.834					
	mTBI-t2	−0.28	0.437					
	All of above	−0.57	0.0002*	84	−11.5	−4.5	+6.5	Bilateral thalamus
	Control-t1	−0.49	0.146					
	Control-t2	−0.90	0.002*	21	+24.5	−28.5	46.5	L-middle frontal gyrus
CSI	mTBI-t1	−0.63	0.053					
	mTBI-t2	−0.98	0.008*	22	+25.0	−21.0	+45.0	L-middle frontal gyrus
	ALL of above	−0.58	0.0002*	23	+24.5	−24.5	+38.5	L-middle frontal gyrus

X, the average ROI correlation coefficient (*r*) and p-value (uncorrected) are also given. The Y and Z represent the peak locations of ROIs in the standard MNI coordinates. Results for datasets with statistically significant (FWER, ≤ p ≤ 0.05) linear correlation were obtained by voxel-wise regression analysis and denoted with *, whereas the results for other datasets were obtained by applying the ROI masks based on the combined CCI and CSI datasets, respectively.
